# F3. A CASE OF LEUKOCYTOSIS ASSOCIATED WITH CLOZAPINE TREATMENT FOR THE MANAGEMENT OF CHRONIC SCHIZOPHRENIA

**DOI:** 10.1093/schbul/sby017.534

**Published:** 2018-04-01

**Authors:** Min-Kyu Song, Won-Myong Bahk, Young Joon Kwon, Bo-Hyun Yoon, Sang-Yeol Lee, Duk-In Jon, Sung-Yong Park, Eunsung Lim

**Affiliations:** 1 Yesan Mental Health Clinic; 2 Yeouido St. Mary’s Hospital, The Catholic University of Korea; 3 College of Medicine, Soonchunhyang University; 4 Naju National Hospital; 5 Wonkwang University, School of Medicine; 6 College of Medicine, Hallym University; 7 Keyo Hospital; 8 Shinsegae Hospital

## Abstract

**Background:**

Clozapine is an atypical antipsychotic drug with therapeutic efficacy through serotonin-dopamine antagonism. It has been used for the management of treatment-resistant schizophrenia and reducing the risk of suicidal behavior. In addition to agranulocytosis and leukopenia, clozapine has also been reported to be associated with other types of blood dyscrasias, including leukocytosis.

We wish to report a case of leukocytosis associated with clozapine treatment in a patient of chronic schizophrenia.

**Methods:**

Our patient is a 48-year-old woman with a diagnosis of schizophrenia since the age of 22. She has a history of numerous hospitalizations and substantial treatment with conservative antipsychotics.

We evaluated her medical and psychiatric history as well as mental status. Then We assumed that she might have treatment-resistant schizophrenia and accordingly commenced treatment with clozapine. At the time of admission, her WBC count was 8.01 × 109/L and ANC was 5110. Clozapine was started at 3rd hospital day. We stopped aripiprazole and gradually increased clozapine. Only 2mg of lorazepam was used in combination with clozapine. Remission was achieved with 450 mg/day of clozapine. WBC on the 1st day of treatment was 12.41 × 109/L (ANC; 9481) and clozapine was 300mg/day. WBC on the 18th day of treatment was 15.32 × 109/L (ANC; 12501), and at that time, her clozapine dosage was 450mg/day. At this point, her vital sign was within normal range and physical examination did not showed any infectious signs. On the 25th day of treatment, WBC count was 22.1 × 109/L and ANC was 17680. However, no general medical condition to explain the leukocytosis was found.

We concluded that her leukocytosis was linked to clozapine, and decided to taper out clozapine despite there being no medical contraindication. After two weeks from starting blonanserin and olanzapine, WBC count normalized. Fortunately, despite replacing the medication, the remission was maintained.

**Results:**

In our case, the increase in white blood cell count with increasing dose of clozapine was evident and did not reveal any other medical cause to explain leukocytosis in the patient. In addition, leukocytosis improved significantly after discontinuing clozapine. Also, there have been reports of cases of leukocytosis associated with clozapine treatment, and the mechanism of leukocytosis has been explained to some extent. Considering these temporal associations, mechanisms, and previous cases, it is reasonable to consider leukocytosis associated with clozapine in this case.

**Discussion:**

This case suggests a temporal relationship between the use of clozapine and leukocytosis. It also shows the rapid resolution of leukocytosis after discontinuation of clozapine. The mechanisms of clozapine-induced leukocytosis may be related to changes in plasmatic concentrations of granulocyte colony-stimulating factor, tumor necrosis factor-α, interleukin (IL)-2 and IL-6 cytokines, which could be stimulated by clozapine. In our case, an increase in WBC count was consistent with an increase in clozapine dose, suggesting a dose-dependent effect. However, the appearance of leukocytosis during clozapine treatment does not mean that clozapine should be discontinued. Nevertheless, the WBC continued to increase steadily, and clinicians inevitably replaced the drugs in consideration of the fact that he was not a general hospital but a psychiatric clinic.

In this paper we have reported a patient of chronic schizophrenia who developed leukocytosis during clozapine treatment. It appears that most of clozapine-associated leukocytosis can be benign medical condition. However, clinicians should be aware of the dangers of other blood disorders such as leukocytosis.

